# Evaluating the integration of HIV self-testing into low-resource health systems: study protocol for a cluster-randomized control trial from EQUIP Innovations

**DOI:** 10.1186/s13063-018-2878-y

**Published:** 2018-09-17

**Authors:** Kathryn Dovel, Frackson Shaba, Mike Nyirenda, O. Agatha Offorjebe, Kelvin Balakasi, Khumbo Phiri, Brooke Nichols, Chi-Hong Tseng, Ashley Bardon, Khumbo Ngona, Risa Hoffman

**Affiliations:** 1 Division of Infectious Diseases, Department of Medicine, University of California Los Angeles, 10833 Le Conte Ave, 37–121 CHS, Los Angeles, CA 90095 USA; 2 Partners in Hope, PO Box 302, Lilongwe, Malawi; 3 David Geffen School of Medicine, University of California Los Angeles, 10833 Le Conte Ave, 37–121 CHS, Los Angeles, CA 90095 USA; 4 Charles R. Drew University of Medicine and Science, Los Angeles, CA USA; 5 HERO, Boston University, Postnet Suite 212, Private Bag X2600, Houghton, 2041 South Africa; 6 Division of General Internal Medicine and Health Services Research, Department of Medicine, University of California Los Angeles, 911 Broxton Ave, 3rd Floor, Los Angeles, CA 90024 USA; 7 Malawian Ministry of Health, Department of HIV/AIDS, Lilongwe, Malawi

**Keywords:** HIV, Sub-Saharan Africa, HIV testing, HIV self-testing, Cost-effectiveness, Randomized control trial

## Abstract

**Background:**

Throughout sub-Saharan Africa HIV-testing rates remain low. Barriers to testing, such as inconvenient service hours and long wait times, lack of privacy, and fear of unwanted disclosure, continue to impede service utilization. HIV self-testing (HIVST) is one strategy that addresses these barriers and has been shown to increase use of HIV-testing when distributed through community-based settings. However, the scalability of HIVST is limited because it has yet to be fully integrated into existing health systems and routine care. To address this gap, we designed a study to test the effect of offering HIVST to routine outpatient department (OPD) clients on uptake of HIV-testing as compared to standard of care and optimized standard of care.

**Methods/design:**

This is a non-blinded, multi-site, cluster-randomized control trial. The health facility is the unit of randomization (cluster). Fifteen facilities were randomized to one of three arms: (1) Standard of care using routine provider-initiated testing and counseling (PITC); (2) Optimized standard of care using optimized PITC defined by additional training, job aids, and monitoring of PITC strategies with OPD providers and support staff; and (3) HIVST defined by HIVST demonstrations for OPD clients, HIVST kit distribution, and private spaces for HIVST kit use and/or interpretation. The primary outcome is the proportion of OPD clients tested for HIV on the day that they accessed OPD services. Secondary outcome measures are the proportion of OPD clients newly identified as HIV-positive and antiretroviral therapy (ART) initiation. Costs and cost-effectiveness will be evaluated. Nested studies will determine the acceptability of facility-based HIVST among OPD clients and health care providers, the presence of adverse events, such as coercion to test or unwanted status disclosure, and a process evaluation to determine feasibility and scale-up of facility-based HIVST for the future.

**Discussion:**

This study protocol tests whether facility-based HIVST can positively contribute to HIV-testing among OPD clients in resource-limited settings. This will be one of the first studies to test the integration of HIVST into facility-based, primary health services in sub-Saharan Africa.

**Trial registration:**

ClinicalTrials.gov, ID: NCT03271307. Registered on 31 August 2017.

Pan African Clinical Trials: PACTR201711002697316. Registered on 1 November 2017.

**Electronic supplementary material:**

The online version of this article (10.1186/s13063-018-2878-y) contains supplementary material, which is available to authorized users.

## Background

Despite increased access to human immunodeficiency virus (HIV)-testing in sub-Saharan Africa, only 45% of HIV-positive individuals know their status [1]. Barriers to testing include limited access to testing services, long wait times, lack of privacy, and fear of unwanted disclosure and stigma [2, 3]. Low use of HIV-testing has significant implications for the 90–90–90 UNAIDS goals that aim for 90% of individuals to know their HIV status, a critical target for curbing the epidemic by 2020 [4]. In order for the first 90 to be reached, new innovations are needed to increase testing.

HIV self-testing (HIVST) is one strategy to increase testing. Self-testing in sub-Saharan Africa is highly acceptable, accurate, and safe [5–8]. When distributed through community-based strategies, self-testing significantly increases the use of testing among hard-to-reach populations such as men and adolescents [6]. However, to date, HIVST strategies have been largely removed from existing health care systems, relying primarily on community-based distribution strategies [5, 6, 9, 10]. Poor integration into national health systems limits the reach and scalability of HIVST, particularly in low-resource settings where Ministries of Health (MOHs) are already strained and have limited capacity for community-based interventions. In addition, linkage to care for those who test HIV-positive through community-based HIVST remains a challenge [11–14]. Strategies to integrate HIVST within existing health systems are needed.

Recent studies have begun integrating HIVST into health systems by using female clients to distribute HIVST kits to male partners in their home [15, 16]. However, this strategy does not address the challenge of linkage to care. It also increases the burden of care for women who are already made largely responsible for the health of their family and uptake of health services [17, 18]. The above limitations may be mitigated by distributing HIVST directly to the intended users at health facilities. Distribution and use of HIVST alongside other facility-based services may improve the scalability of HIVST as well as linkage to antiretroviral therapy (ART) services. Additionally, the use of HIVST in facilities can increase the number of tests performed among facility clients and reduce the burden of testing for facility staff because HIVST does not require direct supervision by a health care worker. In summary, HIVST in facilities may be a strategy to test more people with increased efficiency and decreased costs compared to standard provider-initiated testing and counseling (PITC). A review of the literature shows that no previous randomized control trial (RCT) has been conducted on the distribution and use of HIVST within existing health facilities.

This protocol outlines our proposed methods for a non-blinded, cluster-randomized, matched-controlled trial (cRCT) that tests the effectiveness of integrating HIVST into routine outpatient services in Malawi. We will use three randomization arms: (1) standard of care HIV blood test offered through routine PITC among clients attending the outpatient department (OPD); (2) HIV blood test offered through optimized PITC among OPD clients; and (3) HIVST offered to OPD clients while waiting for routine services (intervention). By combining HIVST with existing facility-based health services, findings may help improve the scalability of HIVST strategies and inform national guidelines for HIV-testing.

## Methods/design

### Objectives

Our overarching objective is to test the feasibility and cost-effectiveness of HIVST among adults receiving OPD health services in Malawi.

### Primary objective

To test whether distributing HIVST at OPD compared to standard of care and PITC will:

- Increase the proportion of OPD clients tested for HIV

### Secondary objective

To test whether distributing HIVST at OPD compared to standard of care and PITC will:

- Be cost-effective
- Increase the proportion of OPD clients newly identified as HIV-positive
- Increase ART initiation among HIV-positive clients tested at OPD

To assess if HIVST at OPD is:

- Acceptable to clients and providers
- Associated with the presence of adverse events such as coercion to test or unwanted status disclosure

### Trial design

**Fig. 1:**
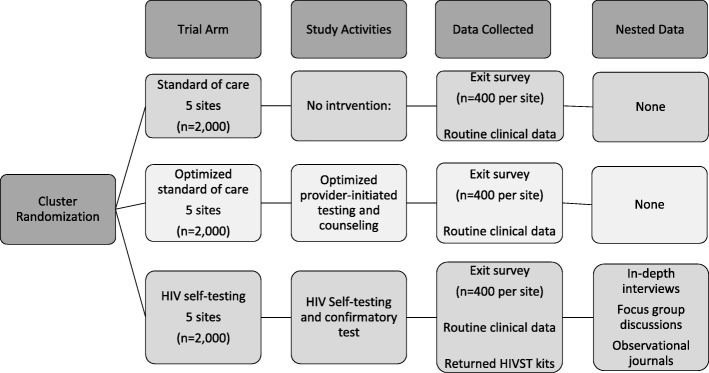
Trial design

We use a non-blinded, cluster-randomized, matched-controlled trial design. The health facility is the unit of randomization (cluster). Fifteen health facilities were randomized in a 1:1:1 allocation to one of three arms: (1) Standard of care (SOC) using routine PITC; (2) Optimized standard of care (OSOC) using optimized PITC defined by additional training, job aids, and monitoring of PITC strategies with OPD providers and support staff; and (3) HIVST (see Fig. 1).

### Trial setting and population

The study will take place in central and southern Malawi. Malawi recently implemented universal treatment for HIV, whereby all clients are eligible for immediate initiation of antiretroviral treatment (ART) [19]. In Malawi, 9% of the adult population are living with HIV, and 42% have tested for HIV in the past year [20]. Study sites include mid- to large-level health facilities that offer outpatient and HIV-testing and treatment services. Sites vary by facility type (hospital/health center), ownership (public/mission), location (rural/urban), and region (central/southern). All adult clients attending OPD services at the time of the study will be eligible for participation. Clients will be excluded if they are under 15 years of age or are not receiving OPD services on the day of recruitment.

### Randomization

Block randomization was used to minimize imbalances between arms. Clusters were blocked by facility type, ownership, and region. Fifteen clusters were allocated 1:1:1 to either the SOC, OSOC, or HIVST arm. Randomization was done by the project statistician using a computer-generated sequence of random numbers. Randomization outputs were shared with the MOH, district-level governance, and medical providers at participating sites in order to maximize transparency of randomization and increase study buy-in.

### Intervention

#### Standard of care arm

All eligible OPD clients attending facilities allocated to the SOC arm will receive standard OPD services. Providers will offer HIV-testing based on their own discretion, as per national guidelines. Current MOH guidelines stipulate that HIV-testing is performed by a certified HIV-testing counselor with blood obtained by fingerstick using Determine 1/2™. A positive test is followed by a confirmatory test using Uni-Gold. Pre- and post-counseling should be provided. Pre-counseling may be provided in a group setting while post-counseling should be provided in a one-on-one format. Guidelines recommend that providers offer HIV-testing to all OPD clients; however, priority is given to clients where there is concern for or confirmation of tuberculosis, clients with symptoms of sexually transmitted infections (STIs), and clients who report high-risk behaviors such as more than one sexual partner in the past 12 months or unprotected sex [19]. No intervention or additional support will be provided at SOC sites. Standard linkage procedures will be followed. All confirmed HIV-positive clients should be documented into the ART linkage register by the HIV-testing counselor and then the client is escorted to the ART clinic to be initiated on ART.

#### Optimized standard of care arm

All eligible OPD clients attending facilities allocated to the OSOC arm will receive standard OPD services. Study personnel will participate in focused efforts to support provider implementation of PITC among OPD clients. Efforts will include PITC refresher trainings, new strategies for PITC implementation to be developed with providers at specific OSOC sites, and intensified monitoring and evaluation of PITC efforts. Standard linkage procedures will be followed as described above.

#### Intervention arm

Clients will receive health education and demonstrations on HIVST while waiting for routine OPD services. Group education will include (1) information about the benefits of testing and treatment, (2) eligibility criteria for use of HIV self-test kits, (3) demonstration on how to use HIV self-test kits, and (4) counseling on what to do if test results are positive or negative. HIV self-test kits will be distributed in OPD waiting areas to interested clients seeking OPD services that day. Because HIVST can only be used for study purposes in Malawi, the study team will distribute HIVST kits, but a facility-based HIV counselor will be present. Clients will be encouraged to only take a kit if they meet the following criteria: (1) are HIV-uninfected or status unknown, (2) have not tested for HIV in the past month, (3) are comfortable using HIVST at the OPD, and (4) are 15 years or older. Clients can opt out from receiving an HIVST kit. Clients may choose to use routine HIV-testing services if they are uncomfortable with HIVST.

HIVST kits will be used in the OPD waiting area, and private spaces will be available for interpreting results. Clients will be encouraged to use and interpret the kit before receiving standard OPD services so they can discuss the test result with their health care provider, if desired. Clients will be asked to return anonymized used or unused HIVST kits to the study team before leaving the OPD so that the study can track distribution, use, and test results.

A trained HIV counselor will be available in the OPD waiting area to answer any questions that arise about how to use the HIVST kit and provide immediate counseling to clients who request additional support. Status disclosure is completely optional. Clients who disclose a positive test result to either health care providers or study staff will be referred to undergo confirmatory testing, using standard HIV-testing services available at the facility. Standard linkage procedures will be followed as described above. See Table 1 for a description of intervention components across arms.

**Table 1:** Intervention components by randomization arm

Randomization arm	Components
Standard of care	● Routine provider-initiated testing and counseling ● No additional support provided
Optimized standard of care	● Optimized provider-initiated testing and counseling ○ Refresher trainings ○ New strategies for PITC implementation ○ Intensified monitoring and evaluation
HIVST intervention	● HIV self-testing ○ Health education and demonstration on HIVST in the OPD waiting area ○ Distribution of HIVST in the OPD waiting area ○ Private spaces at the OPD for reading test results

Legend: *HIVST* HIV self-testing, *OPD* outpatient department, *PITC* provider-initiated testing and counseling

### Study outcomes

The primary study outcome is the proportion of OPD clients tested for HIV in each study arm. We define tested for HIV as using either standard blood tests or HIVST on the day that the clients receive OPD services. The outcome will be measured through participant self-report during an exit survey and confirmed using routine register data. In the HIVST arm, we will also verify testing data by reviewing results of returned HIVST kits.

Secondary outcomes include:

- Cost-effectiveness of HIVST compared to SOC and OSOC, comparing the average cost per test completed and the cost per HIV-positive individual identified between the arms
- Proportion of OPD clients newly identified as HIV-positive using self-report and register data
- The proportion of HIV-positive individuals who link to ART services using self-report and register data
- Client acceptability of the testing strategy using Likert scale questions
- Self-reported adverse events during service utilization (coercion, unwanted disclosure) using Likert scale questions, in-depth interviews, and direct observation

### Sample size considerations

We estimated our sample size assuming a fixed number of clusters (*k*) and an equal number of clusters per arm (*k* = 5). We assumed an equal number of participants per cluster (*n* = 400) for a total sample size of 2000 participants per arm (6000 total). Assuming an overall type I error of 0.05, an intracluster correlation of 0.004, we would expect at least 90% power to detect differences in HIV-testing of 5% in the SOC arm, 10% in the OSOC arm, and 20% in the HIVST arm. This calculation uses the Bonferroni correction to account for multiple comparisons. For secondary outcomes of acceptability based on Likert scale questions, the trial will have 80% power to detect a minimum detectable difference of 0.165 in effect size between any two arms.

### Data collection

Across study arms, data will be collected in two ways: (1) through a one-time exit survey with clients and (2) through digitized routine clinic data, including OPD, ART, and STI registers. Due to clinic workload and clinic flow, it will be impossible to survey all OPD clients (MacPherson 2014). A maximum of 40 clients will be randomly recruited from each facility every day to participate in an exit survey. Recruitment, screening, oral consent, and enrolment for exit surveys will be conducted at the health facility immediately following routine OPD services. Any clients who report testing HIV-positive that same day, or intending to be tested for HIV that same day, will complete a written consent and personal identifiers will be collected. Identifiers will be linked to routine clinical data at the health facility and used to assess ART initiation (for those who tested HIV-positive) and actual use of HIV-testing (for those who wanted to test that same day).

The exit survey was designed in English and translated into Chichewa, the local language. Back translation was completed by an independent person to check and verify question meanings. Exit surveys will be conducted by trained research assistants and answers recorded using Samsung tablets. The survey was programmed using SurveyCTO software (http://www.surveycto.com). Domains in the survey include: sociodemographic; previous use of HIV services; risk behaviour; services received at the facility that day, including HIV-testing; acceptability of the testing strategy implemented in that arm, and for those tested HIV-positive, plans for ART linkage. We will also ask if the participant was offered HIV-testing that day (either standard testing or HIVST) in order to assess the reach of testing across all arms. Participants who self-report as newly HIV-positive and those who did not test but plan to test later that same day will complete written consent, and personal identifiers will be obtained. Identifiers will be used to assess linkage to care for those who test positive and to determine if those indicating they will test later actually complete a test. Newly diagnosed HIV-positive participants who are not linked to care within 2 months will be traced, and outcomes will be determined (moved, died, initiated at another facility, refused to initiate).

For the HIVST arm, all HIV self-test kits will be pre-labeled with unique, anonymous participant ID numbers. Clients will be asked to place all used and unused self-test kits into an anonymous locked box outside the OPD consultation room immediately following the completion of health services. However, clients who participate in an exit survey will return their test kit at the time of the survey and the unique kit ID will be recorded at that time. Survey participants who chose not to take an HIVST kit will be given a random ID number generated by SurveyCTO. Study teams will record the number of kits distributed each day, the number of kits returned, the result of used kits, and the number of kits not returned (missing kits).

**Fig. 2:**
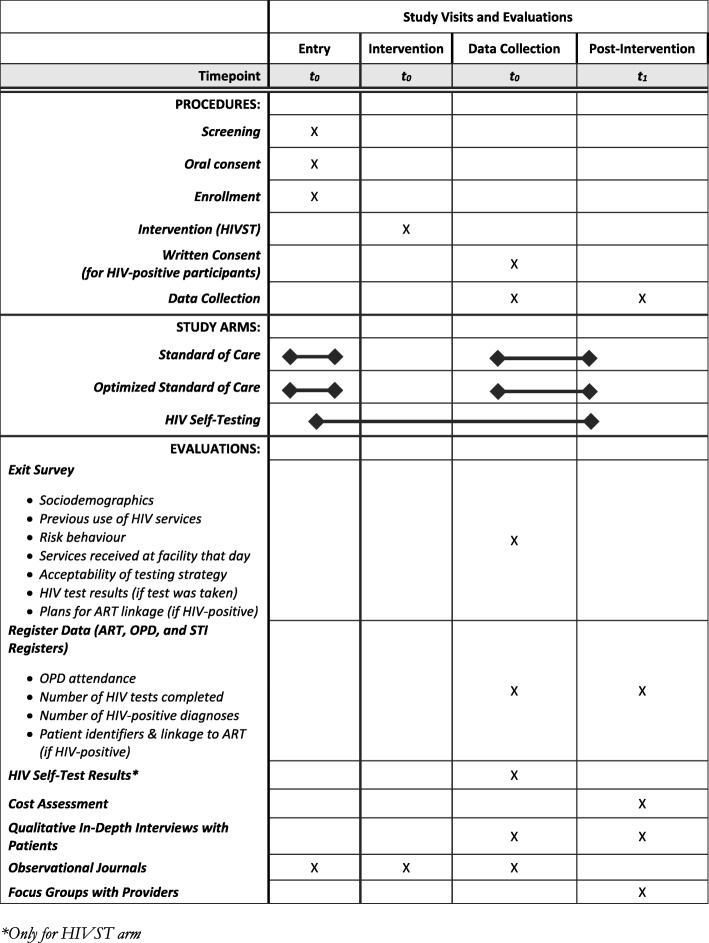
Standard Protocol Items: Recommendations for Interventional Trials (SPIRIT) Figure. *Only for the HIV self-testing (HIVST) arm

Register data will be used to triangulate survey data and will assess OPD attendance, proportion of OPD clients tested for HIV, number of new HIV-positive clients, and the proportion of HIV-positive clients who initiate ART over the study period. Register data will be digitized at the end of each day. See Fig. 2 for a summary of data collection and timelines, following Standard Protocol Items: Recommendations for Interventional Trials (SPIRIT) guidelines.

#### Cost data

A Cost-effectiveness Analysis (CEA) design from a health care perspective using micro-costing methods will be applied to estimate the cost of HIV-testing in all three study arms. An HIV intervention costing instrument, developed by the Health Economics and Epidemiology Research Office (HE^2^RO) and Boston University, will be adopted to collect costing data for the study. Resources to be captured will include: HIV-testing consumables (HIVST kits, standard of care testing supplies), interactions with staff (e.g., counseling, registration), staff training (for standard of care and HIVST), and shared costs and overhead of patient care (building space, utilities, equipment, human resources). The sources of costing data will include facility HIV records, procurement inventories, and hospital personnel records. Replacement costs for all donated equipment will be determined using either recent procurement costs or the current market prices. Cost data will be collected in the local currency, Malawian kwachas. For cost information that is only available in another currency (as with the HIVST kits), the average exchange rate from the US dollar to the Malawian kwacha over the previous year (at time of analysis) will be used.

HIVST kits are procured internationally, and thus their price depends on the exchange rate and recent global fluctuations in the price of HIVST kits. The cost of HIVST kits will, therefore, be varied in a univariate sensitivity analysis where all costs and outcomes remain constant, and the price of self-tests alone is varied.

### Analysis plan

Analyses will be conducted in Stata 14 (Stata Corp., College Station, TX, USA). We will use the Consolidated Standards of Reporting Trials (CONSORT) standards for reporting trial outcomes [21]. We will calculate descriptive statistics, including mean/median, variation (standard deviation, kurtosis), range, and frequency distributions for the demographic and clinical characteristics, overall and by study arm. We will use intention-to-treat (ITT) principles for primary outcome analysis. Mixed-effects models will be used to evaluate the treatment effects on all endpoints, with the fixed effect of treatment assignment and the random effects of study sites, to account for correlations with clusters. This method performs well in situations where the number of observations per cluster is large [22] and for unequal cluster sizes [23].

We will also conduct sensitivity analyses on acceptance of HIV-testing across arms, excluding clients who were not offered testing or were not targeted by the intervention (such as clients with a previous positive test result and clients who tested HIV-negative within the past month). Separate logistic and linear mixed-effects regression models will be developed for each outcome of interest and will include covariates such as demographics (e.g., age, gender), risk behavior (number of sexual partners, condom use), use of HIV services (prior testing, prior test result), and other structural and contextual factors. We will use multiple imputation for imputing missing data not directly related to primary and secondary outcomes [24].

Secondary analyses will examine intervention effectiveness for pre-specified sub-groups of the population, such as men and adolescents, and will include interaction terms where appropriate (sub-group × treatment arm) in the fixed effects. These models will also assess the effect-modification of the intervention effect by facility type.

Cost will be estimated using data collected through the micro-costing strategy described above and utilizing a cost-modeling tool developed by HE^2^RO and Boston University. The incremental cost of identifying one additional new positive or providing one additional test will be calculated (the differences in costs divided by the difference in respective measure of effectiveness) between all study arms. This incremental analysis will be further stratified by level of health facility to see if the cost per outcome achieved differs by facility type.

Secondary outcomes will be explored with the proportions of OPD clients tested HIV-positive, and, of those positive, linked to care. Other outcomes such as client acceptability and potential adverse events (coercion, unwanted disclosure) will be explored, and only those tested will be included in the analysis.

### Nested studies

A series of nested studies will be conducted within the HIVST arm to qualitatively assess acceptability, feasibility, and unintended outcomes of the facility-based HIVST intervention.

#### Qualitative data with clients

A random subset of 60 participants from the HIVST arm (30 men and 30 women) will participate in an in-depth interview to understand client experiences with HIVST at OPD clinics. Respondents will be stratified by facility, sex, use of self-testing, and HIV status. Participants will be asked about their experience with HIVST at the OPD waiting area, including concerns about privacy, problems performing or interpreting the test, and any perceived harms or benefits resulting from self-testing at the OPD clinic. Participants will also be asked about any suggestions or recommendations to improve the distribution and use of HIVST kits at health facilities. Interviews will be conducted by a trained interviewer who is matched by sex with the participant (male-male, female-female). Interviews will be digitally recorded, transcribed, and translated into English.

Observational journals will be collected by the study team and will be used to further understand unintended consequences of the HIVST intervention. Observational journals are intended to capture the content of social interactions; what people say and how they interact with one another in everyday settings, which can be quite different from what they may say during an in-depth interview or survey [25]. A study team member will be assigned to observe clients in the OPD waiting area during the HIVST intervention, taking detailed field notes. Notes will be transcribed into journals at the end of each day. Journals will be written in English. Similar methods have been described elsewhere [26–29].

Interviews and observational journals will be coded and analyzed using similar methods. An a-priori codebook will be developed based on existing literature and will be modified by investigators after reviewing 10 transcripts. Two independent coders will code all data in Atlas.ti using thematic analyses [30] and add new codes as needed [31]. Discrepancies in coding and the addition of new codes will be discussed and resolved. Final codes will be grouped into overarching themes and reported using verbatim quotes that represent key themes.

#### Focus groups with providers

Focus group discussions (FGDs) will be conducted with health care providers in the HIVST arm to better understand provider perceptions and acceptability of HIVST at OPD clinics. Two FGDs will be conducted per facility (10 FGDs total). Focus groups will assess the perceived feasibility of the intervention from a health systems perspective and recommendations for improving the distribution and use of HIVST kits within facilities. Discussions will provide insight into the feasibility of incorporating HIVST into OPD clinics as part of routine care, a critical step towards including facility-based HIVST strategies in national guidelines. All FGDs will be audio-recorded, transcribed, and translated into English. Data will be analyzed with the same methods described above.

#### Implementation log sheet

We understand that an integrated HIVST intervention at OPD clinics has not been done before, and concerns regarding implementation are likely to occur. To track concerns and solutions, the study team will keep a log sheet documenting concerns, solutions, and immediate and mid-term impact of solutions. Data sources for the log sheet include daily reports from the study team in the field, supervision reports, and feedback from key stakeholders such as providers, the MOH, or clients themselves. Findings may influence how similar interventions are implemented in the future.

## Discussion

HIV self-testing offers a promising solution to reach the first and second 90–90–90 UNAIDS goals. However, new, innovative strategies that integrate HIVST into routine health systems are needed if the technology is to be scaled at a national level in resource-limited settings. In this paper, we describe the protocol of a cRCT that will test the effectiveness of an integrated HIVST strategy within routine OPD services in Malawi.

We differentiate our study from other HIVST studies in several ways. First, we will promote distribution and use of HIVST alongside other routine outpatient services. Other studies have shown that incorporating HIV-testing into other primary health services, such as screening for hypertension, increases uptake [32–34]. We build on this work by offering testing to patients accessing outpatient health services in Malawi. Other studies have also used health facilities as distribution points for HIVST, whereby facility clients receive self-test kits to give to their partners at home [15, 16]. Our study will be one of the first to directly target the desired HIVST user and encourage testing at health facilities as part of non-HIV-related health services. Facility-based HIVST may improve linkage to care since testers are already at the health facility, a known limitation of other HIVST strategies [11–14].

Second, this study will be one of the first to measure actual use of HIVST test kits as opposed to self-reported use [6, 15, 35]. We will also be able to link HIVST results with client demographics, HIV risk, and previous use of HIV-testing services. A study in Malawi plans to measure HIVST usage by collecting used kits from male partners who link to health facilities [16]; however, this strategy will be unable to determine if unlinked partners ever used HIVST. Based on the design of our study, we will collect all used and unused kits as participants leave the health facility, allowing us to be certain of HIVST uptake among the target population. A possible limitation will be missing HIVST kits due to clients wanting to take them home for later use or to give to a family member or friend. However, we anticipate this will be a small proportion of total kits distributed.

One concern with any trial testing new diagnostic strategies is the risk of adverse events. Potential adverse events for integrated HIVST at OPD clinics include coercion to test, unwanted status disclosure, and client’s feeling uncomfortable with the intervention, potentially resulting in avoidance of HIV-testing and/or OPD services in the future. Measurement of adverse events is an important secondary outcome of the study.

The study has several limitations. The trial design may be sensitive to the Hawthorne Effect [36]. Based on participation in the exit survey, clients may be more aware of the importance of HIV-testing and thus more likely to test even after receiving all OPD services for that day, potentially reducing the effect of the HIVST intervention against the SOC and OSOC arms. Second, survey clients are recruited at random and may not fully represent the OPD client population. We will adjust for this by controlling for enumerator effects. These limitations will be noted in study findings.

The UNAIDS 90–90–90 goals are lofty targets that require new, scalable technology. HIV self-testing can contribute to targets, but additional strategies to improve HIVST scalability are needed. Our study is one of the first to test the integration of HIVST in routine, facility-based, primary health services. Results from the study will provide important information on facility-based HIVST strategies that can be used to inform HIV-testing policies in sub-Saharan Africa (Additional file 1).

### Trial status

This manuscript was developed using study protocol version 1.4, 16 August 2017, for aim 1 of “Use of HIV Self-Test Kits to Increase Identification of HIV-Infected Individuals and Their Partners.” Recruitment and enrollment began on 18 September 2017. Data collection is expected to be completed by May 2018.

## Additional file

Additional file 1: Standard Protocol Items: Recommendations for Interventional Trials **(**SPIRIT) 2013 Checklist: recommended items to address in a clinical trial protocol and related documents. (DOCX 52 kb)

## Abbreviations

ART: Antiretroviral therapy
CEA: Cost-effectiveness Analysis
cRCT: Cluster-randomized, matched-controlled trial
FGD: Focus group discussion
HIV: Human immunodeficiency virus
HIVST: HIV self-testing
ITT: Intention-to-treat
MOH: Ministry of Health
OPD: Outpatient department
OSOC: Optimized standard of care
PITC: Provider-initiated testing and counseling
RCT: Randomized control trial
SOC: Standard of care
STI: Sexually transmitted infection
UNAIDS: Joint United Nations Programme on HIV/AIDS
USAID: United States Agency for International Development

## References

[1] Joint United Nations Programme on HIV/AIDS (UNAIDS). The Gap Report. Geneva: UNAIDS; 2014.

[2] Musheke M, Ntalasha H, Gari S, Mckenzie O, Bond V, Martin-Hilber A, Merten. A systematic review of qualitative findings on factors enabling and deterring uptake of HIV testing in Sub-Saharan Africa. BMC Public Health. 2013;13(1):220. 10.1186/1471-2458-13-220 PMC3610106 23497196

[3] Meremo A, Mboya B, Ngilangwa D, Dulle R, Tarimo E, Urassa D, Michael E, Gibore N, Mpondo B, McHonde G, et al. Barriers to accessibility and utilization of HIV testing and counseling services in Tanzania: experience from Angaza Zaidi programme. Pan African Medical Journal. 2016;23

[4] Kay ES, Batey DS, Mugavero MJ (2016). The HIV treatment cascade and care continuum: updates, goals, and recommendations for the future. AIDS Res Ther.

[5] Choko AT, Desmond N, Webb EL, Chavula K, Napierala-Mavedzenge S, Gaydos CA, Makombe SD, Chunda T, Squire SB, French N (2011). The uptake and accuracy of oral kits for HIV self-testing in high HIV prevalence setting: a cross-sectional feasibility study in Blantyre, Malawi. PLoS Med.

[6] Choko AT, MacPherson P, Webb EL, Willey BA, Feasy H, Sambakunsi R, Mdolo A, Makombe SD, Desmond N, Hayes R (2015). Uptake, accuracy, safety, and linkage into care over two years of promoting annual self-testing for HIV in Blantyre, Malawi: a community-based prospective study. PLoS Med.

[7] Ng OT, Chow AL, Lee VJ, Chen MI, Win MK, Tan HH, Chua A, Leo YS (2012). Accuracy and user-acceptability of HIV self-testing using an oral fluid-based HIV rapid test. PLoS One.

[8] Zachary D, Mwenge L, Muyoyeta M, Shanaube K, Schaap A, Bond V, Kosloff B, de Haas P, Ayles H. Field comparison of OraQuickW ADVANCE rapid HIV-1/2 antibody test and two blood-based rapid HIV antibody tests in Zambia. BMC Infect Dis. 2012;183 10.1186/1471-2334-12-183 PMC3475053 22871032

[9] MacPherson P, Lalloo DG, Webb EL, Maheswaran H, Choko AT, Makombe SD, Butterworth AE, van Oosterhout JJ, Desmond N, Thindwa D (2014). Effect of optional home initiation of HIV care following HIV self-testing on antiretroviral therapy initiation among adults in Malawi: a randomized clinical trial. JAMA.

[10] Martinez Perez G, Cox V, Ellman T, Moore A, Patten G, Shroufi A, Stinson K, Van Cutsem G, Ibeto MI (2016). Know that I do have HIV but nobody saw me: oral HIV self-testing in an informal settlement in South Africa. PLoS One.

[11] Cherutich P, Kurth A, Musyoki H, Kilonzo N, Maina W. HIV self-testing in sub-Saharan Africa: strategies to enhance and measure linkage to care. Retrovirol: Res Treat. 2014;6:23–8.

[12] Johnson CC, Kennedy C, Fonner V, Siegfried N, Figueroa C, Dalal S, Sands A, Baggaley R (2017). Examining the effects of HIV self-testing compared to standard HIV testing services: a systematic review and meta-analysis. J Int AIDS Soc.

[13] van Rooyen H, Tulloch O, Mukoma W, Makusha T, Chepuka L, Knight LC, Peck RB, Lim JM, Muturi N, Chirwa E, Taegtmeyer M (2015). What are the constraints and opportunities for HIVST scale-up in Africa? Evidence from Kenya, Malawi and South Africa. J Int AIDS Soc.

[14] Walensky RP, Bassett IV (2011). HIV self-testing and the missing linkage. PLoS Med.

[15] Thirumurthy H, Masters SH, Mavedzenge SN, Maman S, Omanga E, Agot K (2016). Promoting male partner HIV testing and safer sexual decision making through secondary distribution of self-tests by HIV-negative female sex workers and women receiving antenatal and post-partum care in Kenya: a cohort study. The Lancet HIV.

[16] Choko AT, Kumwenda MK, Johnson CC, Sakala DW, Chikalipo MC, Fielding K, Chikovore J, Desmond N, Corbett EL (2017). Acceptability of woman-delivered HIV self-testing to the male partner, and additional interventions: a qualitative study of antenatal care participants in Malawi. J Int AIDS Soc.

[17] Dovel K, Yeatman S, Watkins S, Poulin M (2015). Men’s heightened risk of AIDS-related death: the legacy of gendered HIV testing and treatment strategies. AIDS.

[18] Mills E, Beyrer C, Birungi J, Dybul M (2012). Engaging men in prevention and care for HIV/AIDS in Africa. PLoS Med.

[19] Ministry of Health [Malawi] (2016). Clinical management of HIV in children and adults.

[20] National Statistics Office, ICF International (2016). Malawi demographic and health survey 2015–16: key indicators report.

[21] Strauss A, Corbin K (1994). Grounded Theroy methodology. Handbook of qualitative research.

[22] Heo M, Leon AC (2005). Comparison of statistical methods for analysis of clustered binary observations. Stat Med.

[23] Heo M, Leon AC (2005). Performance of a mixed effects logistic regression model for binary outcomes with unequal cluster size. J Biopharm Stat.

[24] Little R, Rubin D (2002). Statistical analysis with missing data.

[25] Watkins SC (2004). Navigating the AIDS epidemic in rural Malawi. Popul Dev Rev.

[26] Angotti N, Sennott C. Implementing “insider” ethnography: lessons from the conversations about HIV/AIDS project in rural South Africa. Qual Res. 2015;15(4):437–53. 10.1177/1468794114543402 PMC4593513 26451131

[27] Conroy Amy, Yeatman Sara, Dovel Kathryn (2013). The social construction of AIDS during a time of evolving access to antiretroviral therapy in rural Malawi. Culture, Health & Sexuality.

[28] Tavory I, Swindler A (2009). Condom semiotics: meaning and condom use in rural Malawi. Am Sociol Rev.

[29] Watkins S, Swindler A (2009). Hearsay ethnography: conversational journals as a method for studying culture in action poetics. Amst.

[30] Boyatzis R (1998). Transforming qualitative information: thematic analysis and code development.

[31] Fereday J, Muir-Cocharne E (2006). Demonstrating rigor using thematic analysis: a hybrid approach to inductive and deductive coding and theme development. Int J Qual Methods.

[32] Odeny TA, Penner J, Lewis-Kulzer J, Leslie HH, Shade SB, Adero W, Kioko J, Cohen CR, Bukusi EA. Integration of HIV care with primary health care services: effect on patient satisfaction and stigma in rural Kenya. AIDS Res Treat. 2013:485715. 10.1155/2013/485715 PMC3664481 23738055

[33] Van Rie A, Clouse K, Hanrahan C, Selibas K, Sanne I, Williams S, Kim P, Bassett J (2014). High uptake of systematic HIV counseling and testing and TB symptom screening at a primary care clinic in South Africa. PLoS One.

[34] Duffy M, Ojikutu B, Andrian S, Sohng E, Minior T, Hirschhorn LR (2017). Non-communicable diseases and HIV care and treatment: models of integrated service delivery. Tropical Med Int Health.

[35] Masters SH, Agot K, Obonyo B, Napierala Mavedzenge S, Maman S, Thirumurthy H (2016). Promoting partner testing and couples testing through secondary distribution of HIV self-tests: a randomized clinical trial. PLoS Med.

[36] McCambridge J, Witton J, Elbourne DR (2014). Systematic review of the Hawthorne effect: new concepts are needed to study research participation effects. J Clin Epidemiol.

